# Low Angle Ring Illumination Stereomicroscopy (LARIS) Method for High-Contrast Imaging of *Drosophila* Compound Eyes

**DOI:** 10.21769/BioProtoc.5590

**Published:** 2026-02-05

**Authors:** Jukta Biswas, Ankur Kumar, Anand K. Singh

**Affiliations:** Department of Biology, Indian Institute of Science Education and Research Tirupati, Yerpedu, Tirupati, Andhra Pradesh, India

**Keywords:** Eye development, Ommatidia, Optical imaging method, Phenotypic screening, Neurodegeneration

## Abstract

The compound eyes of *Drosophila* are widely used to gain valuable insights into genetics, developmental biology, cell biology, disease biology, and gene regulation. Various parameters, such as eye size, pigmentation loss, formation of necrotic patches, and disorientation, fusion, or disruption of ommatidial arrays, are commonly assessed to evaluate eye development and degeneration. We developed an improved imaging method named low-angle ring illumination stereomicroscopy (LARIS) to capture high-contrast images of the *Drosophila* compound eye. Different optical alignments were tested to capture the fly compound eye image under the stereomicroscope; the highest contrast with minimal reflection was achieved through the LARIS method. The images captured using LARIS clearly showed ommatidial fusion, disorientation, and pigmentation loss, which were hardly visible with a conventional imaging method in the degenerating compound eyes of *Drosophila*. In addition to its research applications, this protocol is cost-effective due to the low expenses associated with supplies and equipment. We anticipate that LARIS will facilitate high-contrast imaging of the compound eyes in *Drosophila* and other insects.

Key features

• Low-angle ring illumination stereomicroscopy (LARIS) is an improved optical alignment for high-contrast imaging of *Drosophila* compound eyes.

• LARIS is a simple, inexpensive, and robust method with additional advantages over existing SEM methods to capture high-contrast microscopic images of the *Drosophila* eye.

• LARIS can be easily implemented across laboratories and used as a low-cost teaching/research tool.

## Graphical overview


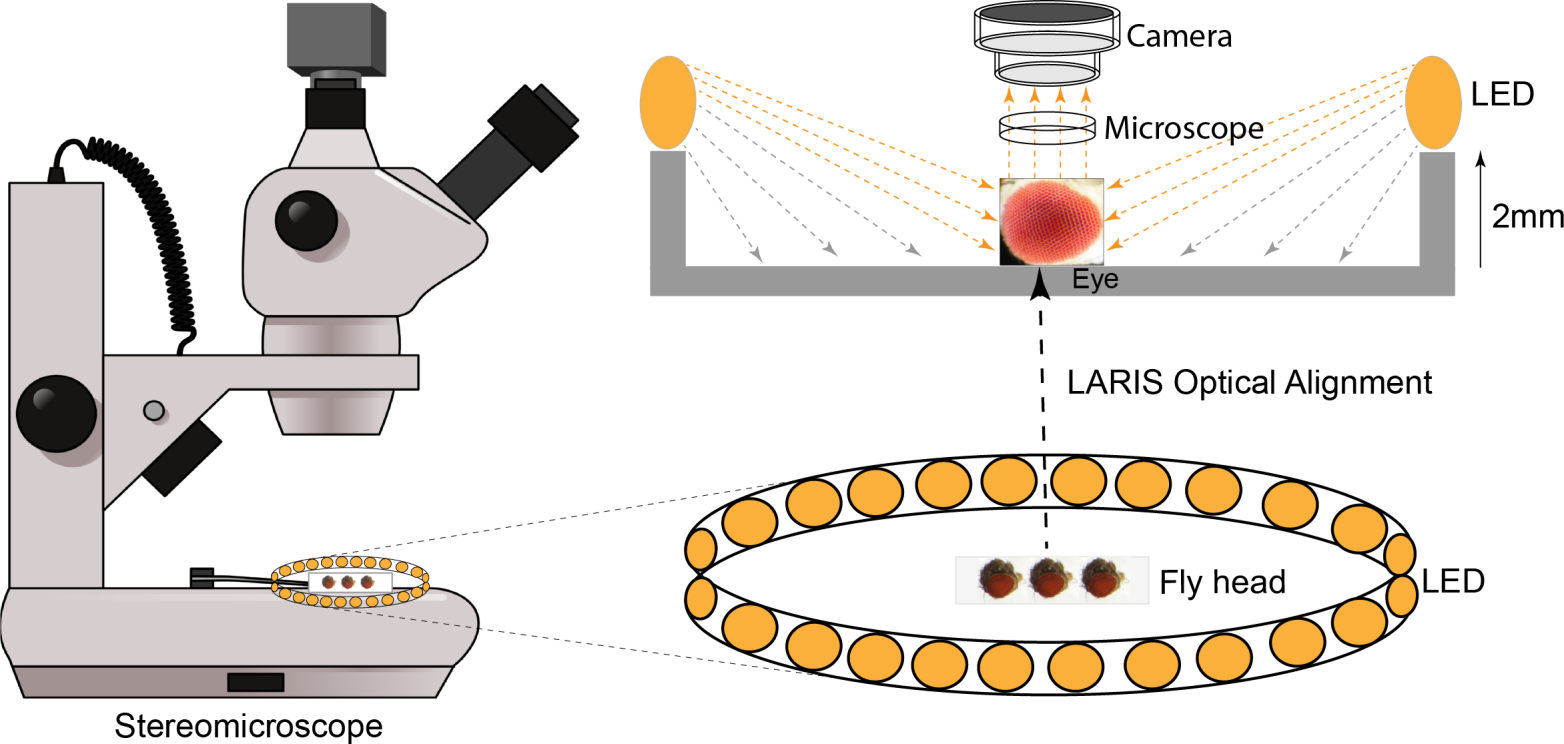


## Background

The *Drosophila* compound eye is comprised of approximately 750 ommatidia that are packed into a remarkably ordered three-dimensional ommatidial array covered with transparent cuticles secreted by cone cells [1]. This layer scatters light, which makes it challenging to capture high-contrast eye images using a stereomicroscope equipped with a standard illumination system. Analysis of such images by a trained researcher or an automated image analysis system can only give an approximation with the possibility of misinterpretation [2,3]. Scanning electron microscopy (SEM) is a popular technique for high-resolution imaging of the eye, but it requires imaging skills and resources [4,5]. Furthermore, SEM images provide no information about pigmentation alteration in the adult fly eye. Nail polish imprint is another high-contrast imaging technique to image the molds of the fly eye, but this technique requires hand skill and does not provide pigmentation details [6,7].

**Figure 1.**
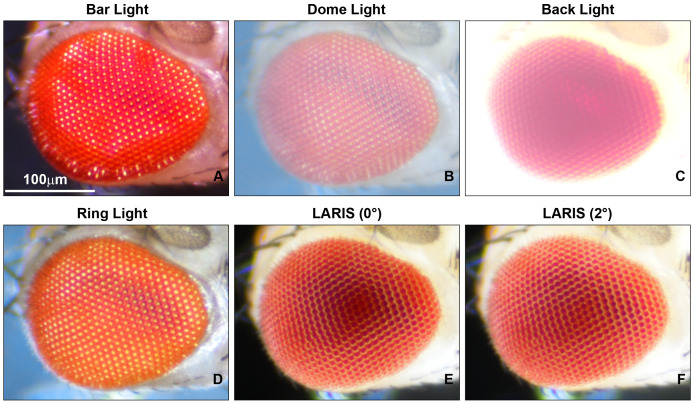
Microphotographs of an adult *Drosophila* compound eye under a simple stereomicroscope with different illumination methods. (A) bar light, (B) dome light, (C) back light, (D) ring light, (E) low-angle ring illumination stereomicroscopy (LARIS) (0°), and (F) LARIS (2°), as mentioned above each panel. All panels are the same sample imaged sequentially at the same magnification without remounting. Scale bar, 100 μm.

To fix this problem, we tested different light sources, including a bar light, a gooseneck light, a diffused/straight backlight, and a ring light to illuminate the eye under a stereomicroscope. Images of bar- or gooseneck light–illuminated eyes had considerable glare with shaded regions (Figure 1A). The dome light alignment, which provided even and diffused light, generated less glare with slightly improved image contrast (Figure 1B). The transmitted light source provided some structural details, but the contrast was poor (Figure 1C). The ring illuminator, fitted around the objective lens of the stereomicroscope, provided a cylinder of light, illuminating the eye from the top. It generated an image with no unwanted shades but an intense glare on the ommatidia (Figure 1D). Also, the ring illumination images provided better contrast but lacked details of the ommatidial structure. We removed the ring illuminator from the objective lens assembly and positioned it closer to the fly on the stereobinocular stage. The glare disappeared when the light source and fly were at the same plane, and a high-contrast ommatidial structure appeared (Figure 1E). However, the central part of the eye lost its contrast because of less illumination than in the peripheral regions of the curved eye surface. This issue was resolved by lifting the ring illuminator approximately 2° upward to the eye position. This improved optical alignment—called low-angle ring illumination stereomicroscopy (LARIS)—did not generate any glare and equally illuminated all parts of the eye, thereby providing a high-contrast image of the entire eye (Figure 1F). In this approach, the position of the ring light source is only slightly above the sample in the LARIS alignment so that the camera receives only the scattered light from the curved surface of the eye. This makes the top surface of each ommatidium slightly brighter than its basal areas, which creates high contrast between neighboring ommatidia and allows distinct visualization of each ommatidium (Figure 1F).

The LARIS method provides better contrast of ommatidial structure, orientation, and pigmentation status than what is obtained by conventional imaging methods of the *Drosophila* compound eye. A comparative analysis of different approaches used in eye imaging of *Drosophila* is given in Table 1. Here, we provide a detailed protocol for the LARIS method to image compound eyes of adult flies or other insects.

**Table 1.** Comparison of LARIS with other imaging methods for the adult *Drosophila* compound eye

Features	Conventional	SEM	Nail polish	LARIS
Contrast	Low	Very high	High	High
Pigmentation record	Yes	No	No	Yes
Sample processing	None	Yes	Yes	None
Cost effectiveness	Low	Very high	Low	Low
Assay timing	Low	Very high	High	Low
Skills and resources	Low	High	Low	Low

## Materials and reagents

**Biological materials**

1. Adult flies (3 days old) of desired genotypes; we used *GMR-GAL4/+; +/+, GMR-GAL4/+; UAS-HttQ138-mRFP/+*, and *GMR-GAL4/+; UAS-FUS^WT^/+* lines for adult eye imaging in this study.

**Laboratory supplies**

1. Glass slide (Blue Star microslide, 75 mm × 25 mm)

2. Transparent nail polish (Shade-40)

3. Fine forceps (Dumont Tweezer, catalog number: 72701-D)

4. Dissection needle (Syringes 31G)

5. Round synthetic paintbrush (Round paintbrush Size 4)

6. Ether (SRL, catalog number: 25049)

7. Etherizer (Tarson, catalog numbers: 630020 and 500043)

## Equipment

1. Stereomicroscope (Nikon, model: SMZ800N)

2. Ring illuminator (AmScope LED-144B-ZK Black 144 PCS Adjustable LED Ring Light)

3. Microscope camera (Nikon, model: DS-Fi3)

## Software and datasets

1. NIS, Elements Imaging Software, Basic Research (BR)

2. Fiji/ImageJ software (NIH, USA) (Java 6, available at imagej.nih.gov/ij)

## Procedure

**A. Sample preparation**

**Figure 2.**
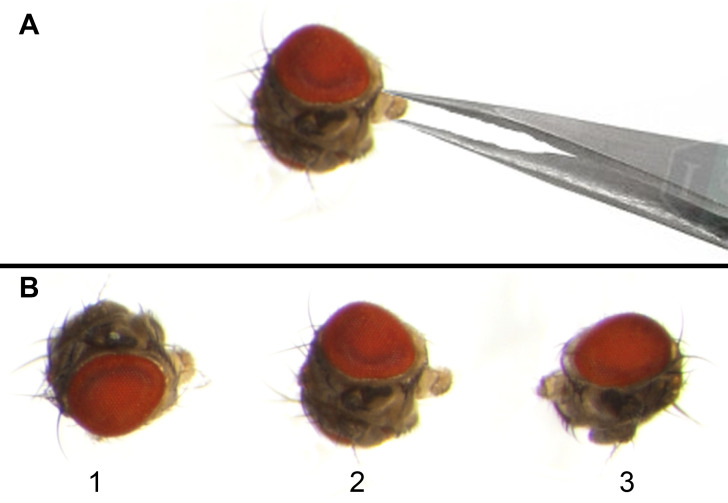
Adult *Drosophila* eye mounting on a glass slide for imaging. (A) Forceps holding the mouth part of the adult fly head so that one eye region can be dipped in nail polish and then attached to the glass slide, while the second eye remains clearly visible under the microscope. (B) Microphotographs of adult *Drosophila* heads (1–3) placed on the mid part of the glass slide for eye imaging. Eyes should be mounted with a proper gap so that the imaging of one eye will not interfere with that of neighboring eyes.

1. Set up the egg laying of desired genetic crosses of parental flies (5 male and 5 female) and allow them to lay eggs for 12 h in vials with standard cornmeal food.

2. Transfer the parental flies to another food vial and culture the F1 (first filial generation) larvae and pupae at 24 °C with 12/12 h light/dark cycles in an incubator until the flies emerge.

3. Collect 3-day-old adult F1 flies and transfer them to an empty clean vial for 10–20 min to allow them to remove food debris particles from their eyes, if attached.

4. Transfer 3–5 male/female flies to the etherizer and incubate them until they stop moving (fully anaesthetized).

5. Transfer the F1 flies immediately from the etherizer to a glass slide and decapitate their heads using a fine dissection needle or forceps by holding the fly at the thorax region with one forceps and detaching the head from the thorax with the help of another forceps.

6. Hold the mouthparts of the fly head with fine forceps and place one side of the fly head, specifically the eye region, on a small drop of nail polish (Figure 2A).

7. Place the fly head on a glass slide with the eye region covered in nail polish adhering to the glass slide, leaving the opposite eye surface facing upward and clearly visible for imaging.

8. Use a fine needle/forceps to align the head to keep the central part of the visible side of the eye in focus with the microscope.

9. Allow the nail polish to dry for 3–5 min. The eye is ready for imaging (Figure 2B).

**B. Microscope alignment**

1. Place a flat plate with a black surface on the stage for taking the images. A white background generates some glare and thus compromises image contrast.

2. Carefully place the slide with eye samples at the center of the stereomicroscope stage.

3. Place the ring illuminator at the stage, keeping the imaging area of the slide at its center. This will ensure equal illumination to the eye from every side.

4. Make a 2–3° angle (slight incline) from the light source to the eye (by putting a 2–3 mm block below the ring illuminator) to stop the formation of a shadow at the center of the compound eye (Figure 1E–F).

5. Set the stereomicroscope at maximum magnification (8× magnification in the SMZ800N).

**C. Image acquisition**

1. Use NIS-Element software for imaging.

2. Bring the sample to the center and adjust the focus to ensure the maximum portion of the eye surface is visible.

3. Concentrate on the central part of the eye while ensuring that as much of the eye as possible remains in focus. Since the eye is a three-dimensional curved structure, a single image cannot entirely bring the eye into focus.

4. Adjust the image brightness by changing the exposure time to 300 ms and gain to 1.0× in the NIS software.

5. Before imaging, perform white balance, keeping the same background without any sample. Do not make any adjustments to settings after white balance.

6. Capture the image with no binning to get a high-resolution image.

**D. Image analysis**

1. Capture 50 eyes of each genotype at a minimum, keeping all the imaging parameters constant to maintain similar brightness and color temperature across genotypes and replicates.

2. Eye defects can be quantified by a degeneration score, which takes into account eye size, the number and size of necrotic patches, pigmentation level, and ommatidial alignment and fusion.

3. Using these parameters, the eye degeneration score can be calculated to illustrate the rescuing or exacerbating effects of the targeted molecule on neurodegeneration in the *Drosophila* compound eye.

## Data analysis

**Figure 3.**
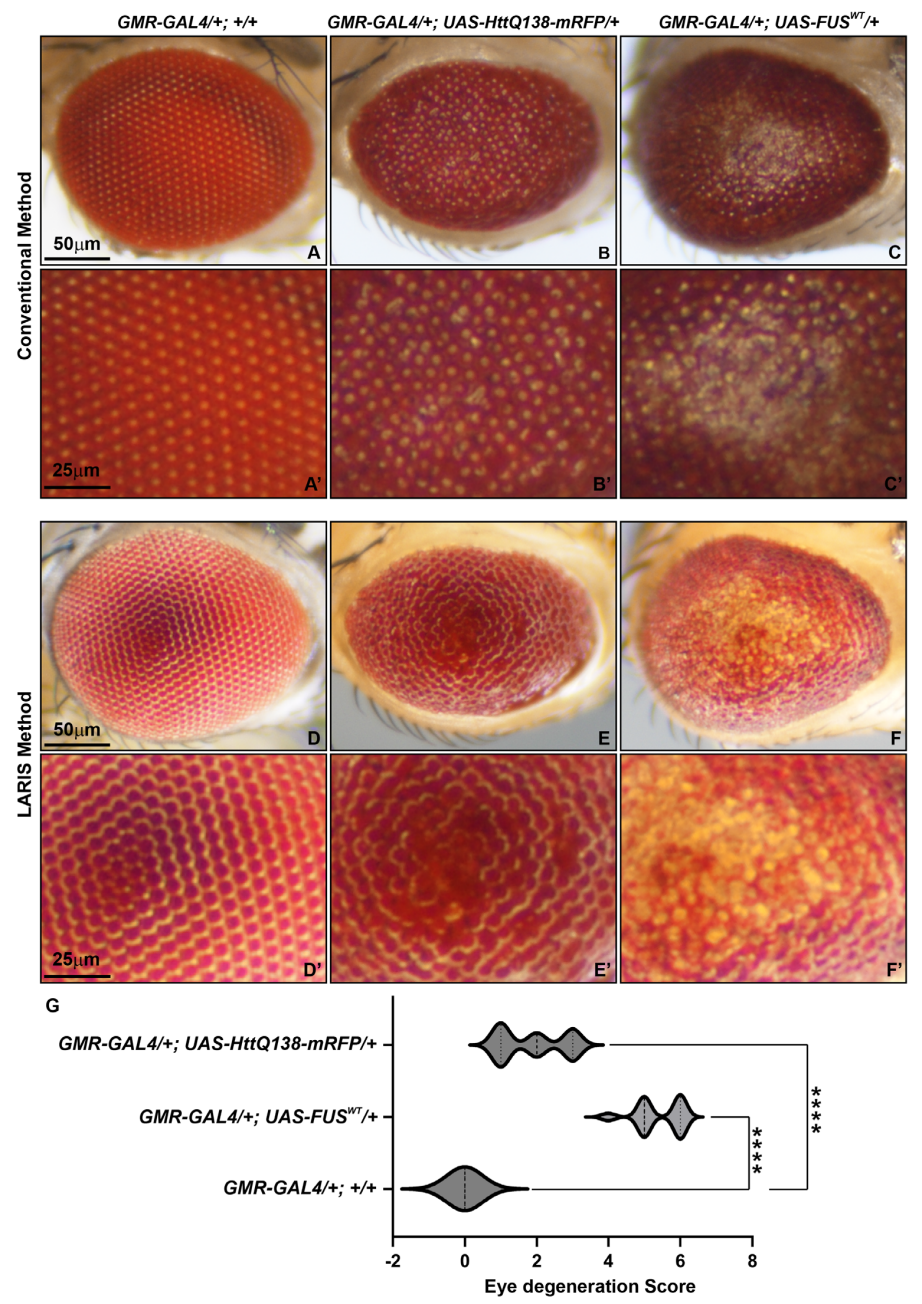
Comparative analysis of conventional and LARIS imaging methods of adult *Drosophila* eyes. Microphotographs of adult *Drosophila* compound eye of *GMR-GAL4/+; +/+* (A, D), *GMR-GAL4/+; UAS-HttQ138-mRFP/+* (B, E), and *GMR-GAL4/+; UAS-FUS^WT^/+* (C, F) using conventional (A–C) or LARIS (D–F) imaging at the same magnification (5×). A magnified view of conventional images (A’–C’) and LARIS images (D’–F’) is shown below their respective images. (G) *Drosophila* compound eye degeneration score, calculated from eye images captured using the LARIS method, showing statistically significant high neurodegeneration in *GMR-GAL4/+; UAS-HttQ138-mRFP/+* and *GMR-GAL4/+; UAS-FUS^WT^/+* lines compared to control flies. An ordinary one-way ANOVA with Sidak’s multiple comparisons test was used to calculate statistical significance. ****p < 0.0001, n = 25 for each genotype (middle line of violin plot indicates the median of the degeneration score).

We compared conventional and LARIS imaging methods to examine the differences in structural details in the eyes of normal or a neurodegeneration model of *Drosophila*. We used *GMR-GAL4/+; +/+* [8] fly line as a control, *GMR-GAL4/+; UAS-HttQ138-mRFP/+* [9] as the Huntington’s disease model, and *GMR-GAL4/+; UAS-FUS^WT^/+* [10] as the ALS disease model (Figure 3). Under conventional imaging, the wildtype eye has only an array of bright dots without enough structural details (Figure 3A, A’). The same eye under LARIS shows distinct ommatidia aligned in rows (Figure 3D, D’). In neurodegeneration lines, where the conventional method provides images with no distinct information on ommatidial structure and arrangement (Figure 3B, B’, C, C’), LARIS images showed ommatidial fusion in *GMR-GAL4/+; UAS-HttQ138-mRFP/+* flies (Figure 3E, E’) and an apparent loss of ommatidial structure at the central part of the *GMR-GAL4/+; UAS-FUS^WT^/+* flies (Figure 3F, F’). The abnormalities in ommatidial structure and arrangement in degenerating eyes are even more distinct in a magnified view of the LARIS image. We quantified the degeneration score by scoring various parameters of eye phenotype [11] and found that the degeneration score of the neurodegeneration lines is significantly higher compared to control flies (Figure 3G). Our study showed that LARIS is a straightforward, reliable, and robust method for capturing high-contrast images of insect compound eyes. This technique is particularly beneficial for imaging the three-dimensional microscopic structures with highly reflective cuticles found in *Drosophila* and other organisms. We believe that LARIS will create new opportunities for high-contrast imaging of insect eyes.

## Validation of protocol

We tested the LARIS method for eye imaging of different genotypes; validation of the protocol is provided in Figures 1 and 3.

## General notes and troubleshooting

**General notes**

1. A standard CO_2_ fly pad can be used in place of ether to anaesthetize the adult flies.

2. The forceps should not touch the eye during decapitation of the head. Some eyes with neurodegeneration shrink within the head region and thus can generate an artifact.

3. Use a tiny drop of nail polish, which is sufficient for attaching one side of the eye to a glass slide. An excess amount may allow the head to get merged in nail polish, creating a layer of nail polish on the imaging eye and thus affecting image quality.

4. A proper alignment of the head on the slide is crucial to focus the image on the central part and thus cover the maximum area of the eye.

5. Keep the ring illuminator at maximum brightness. Adjust the imaging light intensity by adjusting the exposure time to 300 ms and gain to 1.0× in the NIS Elements software.

6. The 2–3 mm block, which is required to make an angle between the ring illuminator and the eye sample, can be made by stacking 2–3 glass slides.

7. White balance is a function used in microscopy and imaging to correct the color temperature of an image, making white objects appear true white by adjusting the red, green, and blue components. This ensures that colors are an accurate representation of the specimen, free from the color cast of the illumination source.

8. The imaging table should be vibration-free. Do not touch the microscope or table during image capturing.

9. Since the *Drosophila* compound eye is a curved structure, getting a complete eye image requires multiple images of the same eye with different focus points. This series of images can be used to create a completely focused eye image by using an extended depth of field (EDF) application in imaging software https://www.nisoftware.net/NikonSaleApplication/Help/Docs-D/eng_d/edf.step.by.step.html.

10. Keep all imaging parameters constant for comparative imaging.

**Troubleshooting**

1. Eyes with complete loss of pigmentation may exhibit some glare with the LARIS method. Keep the ring illuminator parallel to the object (0° angle) to minimize glare.

2. Eyes with a high level of degeneration may invaginate within 20–30 min of incubation after decapitation. To avoid such artifacts in the eye image, perform imaging immediately after mounting.

3. To ensure consistent eye image color intensity, perform white balance with a black background before starting imaging.

## Acknowledgements

We acknowledge Prof. S. C. Lakhotia from Banaras Hindu University, India, for providing the fly stocks and his valuable suggestions on image properties, and Dr. T. Kanagasekaran from the Indian Institute of Science Education and Research, Tirupati, for his description of the microscope optics. We thank Prof. Girish Ratnaparkhi from IISER Pune and Prof. Surajit Sarkar from the University of Delhi for their kind gifts of FUS^WT^ and HttQ138-mRFP lines, respectively. We thank the Science and Engineering Research Board, grant/award number CRG/2022/002767, and the Department of Biotechnology, Ministry of Science and Technology, India, grant/award number BT/RHD/35/02/2006, for funding support.
